# Population Trends of Central European Montane Birds Provide Evidence for Adverse Impacts of Climate Change on High-Altitude Species

**DOI:** 10.1371/journal.pone.0139465

**Published:** 2015-10-01

**Authors:** Jiří Flousek, Tomáš Telenský, Jan Hanzelka, Jiří Reif

**Affiliations:** 1 Krkonoše National Park Administration, Dobrovského 3, CZ-543 01, Vrchlabí, Czech Republic; 2 Institute for Environmental Studies, Faculty of Science, Charles University in Prague, Benátská 2, CZ-128 01, Praha 2, Czech Republic; 3 Institute of Vertebrate Biology, Academy of Sciences of the Czech Republic, v. v. i., Květná 8, Brno, 603 65, Czech Republic; 4 Czech Society for Ornithology, Na Bělidle 34, CZ-150 00, Praha 5, Czech Republic; Universidad de Granada, SPAIN

## Abstract

Climate change is among the most important global threats to biodiversity and mountain areas are supposed to be under especially high pressure. Although recent modelling studies suggest considerable future range contractions of montane species accompanied with increased extinction risk, data allowing to test actual population consequences of the observed climate changes and identifying traits associated to their adverse impacts are very scarce. To fill this knowledge gap, we estimated long-term population trends of montane birds from 1984 to 2011 in a central European mountain range, the Giant Mountains (Krkonoše), where significant warming occurred over this period. We then related the population trends to several species' traits related to the climate change effects. We found that the species breeding in various habitats at higher altitudes had more negative trends than species breeding at lower altitudes. We also found that the species moved upwards as a response to warming climate, and these altitudinal range shifts were associated with more positive population trends at lower altitudes than at higher altitudes. Moreover, long-distance migrants declined more than residents or species migrating for shorter distances. Taken together, these results indicate that the climate change, besides other possible environmental changes, already influences populations of montane birds with particularly adverse impacts on high-altitude species such as water pipit (*Anthus spinoletta*). It is evident that the alpine species, predicted to undergo serious climatically induced range contractions due to warming climate in the future, already started moving along this trajectory.

## Introduction

Climate change ranks among the top drivers of biodiversity changes worldwide [1]. However, severity of its impacts varies over the Earth’s surface with mountain areas being among those under extraordinarily high pressure [2, 3]. To better understand potential consequences of climate change in these areas, it is important to quantify the magnitude of this impact on species’ populations and to determine the species being under the highest risk [4].

Under the circumstances of climate change, montane species typically shift their ranges towards higher altitudes tracking their climatic optima [5–8], although some other drivers might be also involved in such shifts [9]. This pattern of shift was found in vast majority of cases because it is consistent with globally warming temperatures and a gradient of decreasing temperature with altitude [10]. However, space limitations at high altitudes constrain possibilities of montane species to cope with climatic changes and make them particularly vulnerable: in an extreme case, the climatic niche of some of these species can move beyond the mountain tops driving such species ultimately to extinction [11]. Although various studies modelled and predicted such threats for the future conditions [2, 12, 13], empirical evidence for these impacts of the current climate change on montane species remains limited due to the lack of long-term data on species’ distribution and abundance at high altitudes [14].

Here we focused on the impacts of climate change on long-term population trends of birds in a central European mountain range, the Giant (Krkonoše) Mountains, Czech Republic. The Giant Mts. cover an altitudinal range of 1200 m from the low-altitude forests and cultural landscape to high-altitude alpine grasslands above timberline [15]. Quantification of long-term population trends was enabled by the existence of a unique long-term data set collected using a standardized technique by a single observer since 1984 and covering the entire altitudinal gradient in these mountains. We studied the effects of climate change on populations of montane bird species by means of interspecific comparative analysis relating population trends to species-specific traits [16] testing following predictions.

(i) Population trends of species breeding at higher altitudes should be more negative than the trends of species breeding at lower altitudes. Distribution of the species breeding at high altitudes is constrained by mountain tops and thus their populations are more likely to decline under the conditions of warming climate [11]. (ii) Long-distance migrants should have more negative trends than the short-distance migrants and residents because they are more adversely affected by climatic changes on breeding grounds [17] and also adversely affected by habitat deterioration at the wintering and stopover sites [18]. (iii) Species with slow life history strategies should have more negative trends than the species with fast strategies. The fast life history is characterized by potentially high population growth rate [19] enabling a quick recovery of species’ population after an impact of environmental change [20], which is more problematic for the slow life history species making them more sensitive to the impacts of global changes [21]. On the other hand, slow life history species may be more resistant to environmental pertubations making their populations more stable [22, 23]. In addition, the climate change was found to affect central European populations of birds according to the mean temperature in their European breeding ranges, when regional abundance of species breeding in warmer (southern or lowland) regions increase, while species of colder (northern or montane) regions decline [21, 24, 25, 26]. Therefore, (iv) species with lower temperature of European breeding range should have more negative population trends than the species breeding in warmer temperatures.

Population trends of particular species may also be related to upward shifts of their altitudinal ranges caused by warming climate [27]. By shifting to higher altitudes species compensate the adverse impacts of climate change on their populations [28]. Therefore, depending on the altitude of species’ breeding occurrence, we could expect differences in population trends between long- and short-shifting species. Specifically, population trends of species experiencing longer altitudinal range shifts should be less negative than the trends of species showing shorter shifts in the case of species breeding at lower altitudes, but the reverse will be true at higher altitudes. We thus tested this hypothesis as a (v) final prediction.

The aims of this study were (i) to calculate the long-term population trends of particular bird species breeding in the Giant Mts., (ii) to describe the climate change in this mountain range, (iii) to test the predictions formulated above.

## Materials and Methods

### Study area

The study was conducted in the Giant Mts. (Krkonoše), the highest mountain range in the Czech Republic spanning an altitudinal range of more than 1200 m and with the highest peak of 1603 m a.s.l. The range spreads over four altitudinal vegetation belts: submontane, montane, subalpine and alpine [29, 30]. The two lower belts are covered by forests with a timber line at about 1300 m a.s.l.: more or less close-to-nature and autochthonous beech-spruce and mountain spruce forests prevail at higher elevations, whereas most forests at lower elevations are managed mixed and spruce stands [31]. The two upper belts are covered by open habitats of artic-alpine tundra [32]: glacial corries, rocks, alpine and subalpine grasslands, subarctic peatbogs and stands of *Pinus mugo*. Meadows and pastures are also present at lower elevations replacing original forest vegetation in some areas below and around the timber line [33]. Most of the Giant Mts. is protected as a national park and the intensity of human impact decreases from the foothills and valleys with permanent human population towards higher altitudes [31]. See [7] for more details on the study area.

### Climate data

To describe patterns in climate change during the bird breeding season (May-July; note that the breeding starts later in montane environment) in the Giant Mts. over the study period we used data collected at three meteorological stations (S1 Table): Labska bouda (1315 m a.s.l.), Pec pod Snezkou (816 m a.s.l.) and Janske Lazne (650 m a.s.l.). The stations were located in different parts of the national park providing meaningful information about the climatic conditions in the Giant Mts. The data are the mean temperatures over the focal months (May-July) supplied by the Czech Hydrometeorological Institute.

### Bird census

Bird census was approved by the Krkonoše National Park Administration. Since it was based just on observation and hearing of bird individuals without any disturbance, there was no need for approval of animal welfare committee. Birds were counted by JF along ten transects scattered throughout the mountain range covering all altitudinal vegetation belts (see Fig A1 in [7]) annually from 1984 to 2011. Transects contained 6–27 points located in ca 400 m intervals. Each point was visited two times per breeding season (May-July) early in the morning under favourable weather conditions (no rain or fog, no strong wind). During one visit, all birds seen or heard were recorded within 100 m radius around each census point for five minutes. Locations of the points and transects did not change over the course of the study and the dates of the visits varied less than ±7 days between years. Maximum count from both visits was taken as the abundance of a given species at one point in a given year. Such maximum counts are frequently used in studies inferring population trends from annual monitoring data because they are most likely closer to real abundances than, for example, mean counts [21, 34]. These point-abundances were summed to calculate the abundance of every species at each transect in a given year. These annual abundances at the transect level were used for further analysis to estimate the species’ population trends.

### Bird population trends

Population trends were estimated for all but one 51 common bird species whose altitudinal range shifts in the Giant Mts. were investigated in our earlier study [7]. The only exception was black grouse (*Tetrao tetrix*) whose counts were too low (five observations per year on average) and we thus excluded this species from the analysis of trends. Our final dataset thus contained 50 species. Trends were computed in TRIM, a statistical software developed specifically for the analysis of long-term time series data from animal monitoring programmes [35], which is currently among the most frequently used frameworks to infer bird population trends (see e.g. [24, 25, 36–38]). For the analysis we employed log-linear models with Poisson error structure where species’ abundance at particular transects was a response variable (see above) and years (1984–2011) and transects were respective explanatory variables. Moreover, serial correlation and overdispersion were taken into account. As a result, we obtained yearly population indices of particular species and the population trend of a given species was estimated as a slope (with its standard error) of the regression line through the logarithms of the yearly indices. The trend can thus be translated as a logarithm of mean annual population growth rate. Negative values of trends signify population decline, and positive values population increase. These species’ trends and standard errors (S2 Table) were taken for further analysis.

### Species’ traits

For each bird species, we defined following ecological traits (S2 Table):

*Mean altitude of the breeding occurrence* is the mean altitude (m) of the census points in the Giant Mts. where a given species was detected during the breeding season in the time period 1986–1988. These data were taken from our earlier study [7] and provide information about the mean altitude of species’ distribution in the Giant Mts. early after the bird monitoring had started.

*Altitudinal range shift* is the mean annual shift of breeding altitudinal range of a given species between 1986 and 2011. Although altitudinal range shifts of birds in the Giant Mts. were already quantified in our earlier study [7], they had data only from three shorter periods available. Therefore, we decided to use bird census data collected at the annual basis for the purpose of the current study. We adopted the approach described in [39] focusing on shifts in the mean altitude of species’ occurrence. To take the information from each year of the time series into account, we first calculated the mean altitude of occurrence in Giant Mts. for each species in a given year as a weighted mean of altitudes of points occupied by a species with its abundance on these points taken as a weight. These mean altitudes we regressed across years revealing species-specific slopes quantifying the magnitude of mean annual shift of each species over the focal time period. These species-specific slopes were taken as a response variable in further analysis.

*Migration strategy* is based on the information about migratory habits of particular species inferred from ringing recoveries collected over 20th century and published in the Czech Bird Migration Atlas [40]. According to the information in [40] species are classified as (1) residents (wintering in central Europe), (2) short-distance migrants (wintering in Western Europe or Mediterranean region) and (3) long-distance migrants (wintering in sub-Saharan Africa or Asia).

*Life history strategy* was expressed as a first ordination axis from a principal component analysis on species’ values of six life history traits (body mass, egg mass, number of broods per year, laying date, clutch size and incubation length) performed by [41]. This axis ordinated species along a fast-slow life history gradient from “r-selected” to “K-selected” species.

*European climatic niche* was taken from [42] as a mean temperature within species’ European breeding ranges. It was calculated by crossing maps of species’ breeding distribution in Europe [43] with maps of mean temperatures in species-specific breeding season in particular mapping squares. See [42] for more details on its calculation.

### Statistical analysis

We first calculated pairwise correlations among the trait variables (S3 Table). Pearson correlation coefficients were considerably lower than the level indicating that multicollinearity among predictors would be an issue [44]. Therefore, we used all traits for further analysis.

We related bird population trends to species’ traits using linear models with the trend as a response variable, particular traits as explanatory variables and the inverse standard error of trend as a weight to give more weight to species with more accurate trend estimates, which is often used in such studies (see e.g. [24–25]). To test our hypotheses, we first composed a model containing the main effects of all traits as well as the interaction between the mean altitude of breeding occurrence and the altitudinal range shift (full model). To obtain the parameter estimates for the main effects of interacting variables not affected by their interactions, we also fitted the model without interaction term (main effects model).

All explanatory variables were standardized prior to statistical analysis to obtain comparable parameter estimates [45].

Bird species are phylogenetically related to various extent due to common evolutionary history [46]. Therefore, it is necessary to test whether this effect could influence the results of statistical modelling [47]. For this purpose, we tested for the presence of phylogenetic autocorrelation in residuals of the tested models using Moran’s I in the R-package ‘ape’ [48]. Phylogeny of the focal species was extracted from [49].

Annual data on temperatures in the breeding season from particular meteorological stations were regressed across years to reveal the trends over time.

## Results

### Climate change

**Fig 1:**
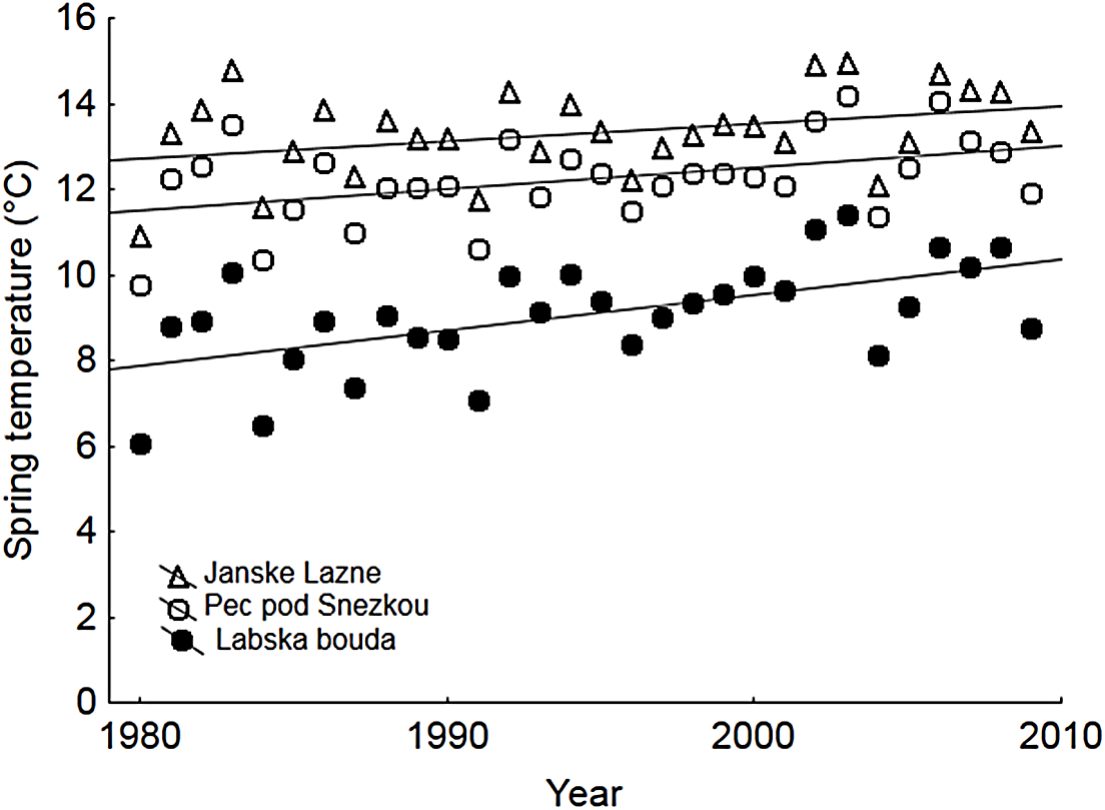
Annual changes of mean temperatures in the Giant Mountains. The temperatures refer to the local breeding season of birds (May-July) and were measured at three meteorological stations (Labska bouda: 1315 m a.s.l.—black circles, Pec pod Snezkou: 816 m a.s.l.—open circles, Janske Lazne: 650 m a.s.l.—open triangles). Solid lines are linear fits depicted for respective data sets.

Temperatures in the bird breeding season increased from 1980 to 2009 according to data from all three stations located in the Giant Mts. (F_1,84_ = 24.27, P < 0.001; Fig 1). Interestingly, the rate of increase was slightly higher at the station located in the highest elevation (Labska bouda: intercept = 7.8, slope = 0.08°C/year, R^2^ = 0.33) than at the two other stations in lower elevations of the Giant Mts. (Pec pod Snezkou: intercept = 11.4, slope = 0.05°C/year, R^2^ = 0.19; Janske Lazne: intercept = 12.7, slope = 0.04°C/year, R^2^ = 0.13) suggesting the climate change is progressing somewhat quicker at higher altitudes, but not significantly so (F_2,84_ = 1.19, P = 0.309).

### Bird population changes

More species had positive (n = 31) than negative trends (n = 19) in our sample. Within the species showing the strongest declines dominated those breeding at the highest altitudes of the Giant Mts.: specialists strictly confined to the alpine habitats near mountaintops such as water pipit (*Anthus spinoletta*) or bluethroat (*Luscinia svecica svecica*), species of alpine grasslands and montane meadows such as skylark (*Alauda arvensis*) or meadow pipit (*Anthus pratensis*), species of rocky outcrops and human buildings such as black redstart (*Phoenicurus ochruros*) or white wagtail (*Motacilla alba*) and species most abundant in shrubby habitats along timberline such as whinchat (*Saxicola rubetra*) or tree pipit (*Anthus trivialis*).

In the interspecific comparative analysis, the full model explained 28.53% of variability in long-term population trends of montane birds (F_6,43_ = 4.26, P = 0.002) and showed that the traits with the significant main effects were the mean altitude of the breeding occurrence and migration strategy, while the altitudinal range shift, life history strategy and European climatic niche were unrelated to the trends (Table 1). However, the mean altitude of the breeding occurrence × altitudinal range shift was significant (Table 1). By deleting the interaction term we obtained the main effects model. It showed the same significant main effects as the previous model (Table 1) but the amount of variability explained by this model was lower—22.65% (F_5,44_ = 3.87, P = 0.005).

**Table 1:** Relationships between long-term population trends of birds breeding in the Giant Mountains (Czech Republic), estimated for the time period 1984–2011, and particular species’ traits as revealed by linear models.

Model term	Full model				Main effects model			
	Estimate	SE	t	P	Estimate	SE	t	P
mean altitude of breeding occurrence	**-0.40**	**0.12**	**-3.40**	**0.001**	**-0.38**	**0.12**	**-3.09**	**0.003**
altitudinal range shift	0.29	0.16	1.85	0.071	0.21	0.16	1.34	0.187
migration strategy	**-0.26**	**0.11**	**-2.39**	**0.021**	**-0.24**	**0.11**	**-2.18**	**0.035**
life history strategy	-0.01	0.12	-0.12	0.905	0.03	0.12	0.27	0.788
European climatic niche	0.01	0.10	0.11	0.914	0.05	0.11	0.43	0.670
mean altitude of breeding occurrence × altitudinal range shift	**-0.35**	**0.16**	**-2.15**	**0.037**	-	-	-	-

Significant results are printed in bold.

See text for definitions of particular trait variables and for more details on the models.

The explanatory variables were standardized to zero mean and unit variance before analysis.

**Fig 2:**
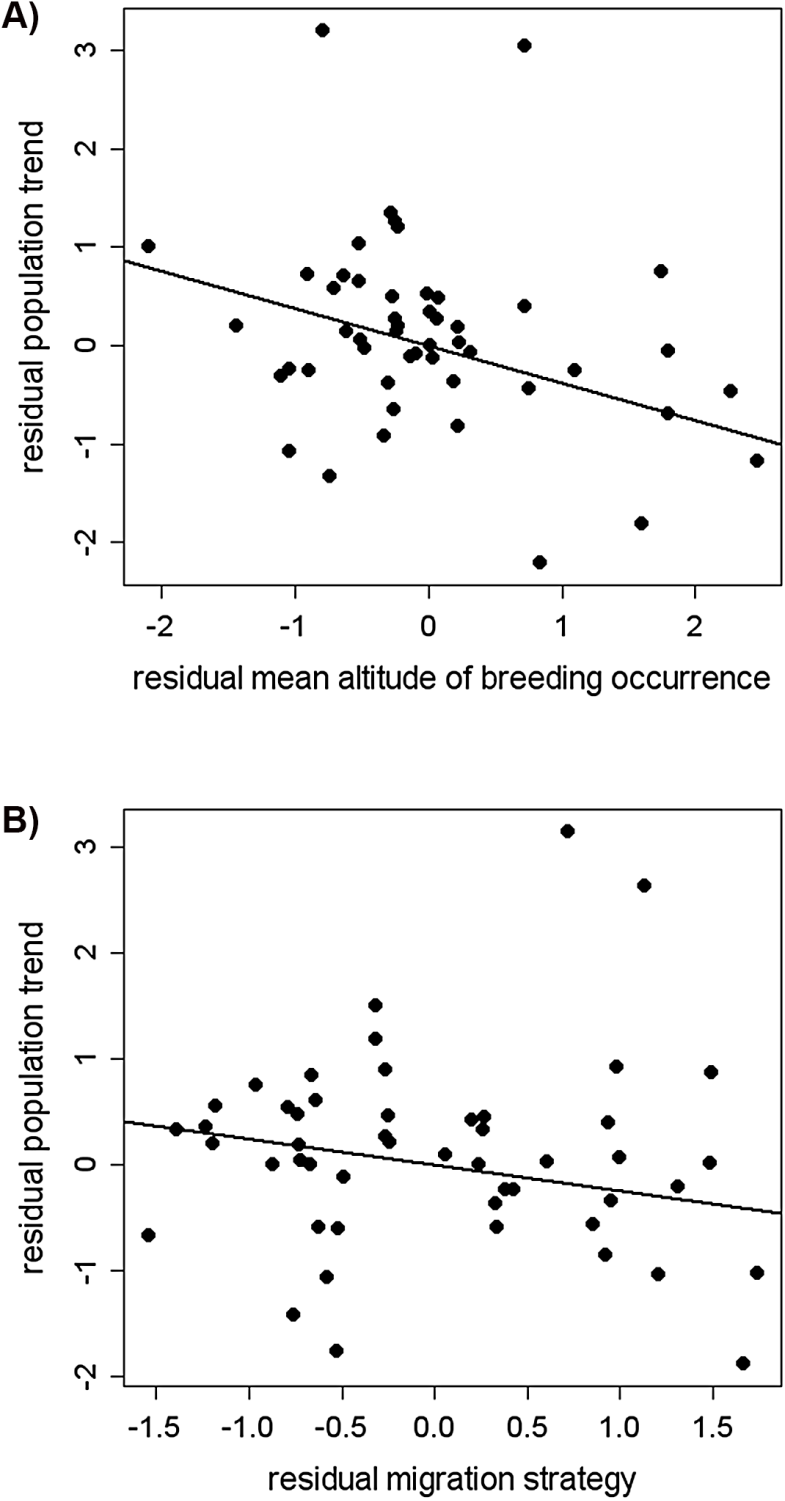
Relationships between long-term population trends of birds breeding in the Giant Mountains and their predictors. The trends were estimated for the time period 1984–2011 and are significantly related to (a) mean altitude of breeding occurrence at beginning of the monitoring period (the higher the value, the higher altitude a given species uses for breeding) and (b) migration strategy (the higher the value, the longer migration route a given species takes) as revealed by the linear main effects model (see text for more details on particular variables and the model). The plots show pure effects of the focal variables after controlling for the effects of all other traits.

The parameter estimates from the main effects model (Table 1) confirmed the observation of population declines in the high-altitude species when the effect of the mean altitude of the breeding occurrence was strongly negative with more negative population trends having the species breeding at higher altitudes (Fig 2A). Concerning the effect of migration strategy, the longer migratory route was associated with more negative trends (Fig 2B). We also found a significantly negative interaction between the altitudinal range shift and the mean altitude of the breeding occurrence (Table 1). It means that the long altitudinal range shift is beneficial for species breeding at lower altitudes, but it is associated with population declines at higher altitudes.

The residuals of both models did not indicate any significant phylogenetic autocorrelation in data (full model: Moran’s I = -0.02, SD = 0.01, p = 0.752; main effects model: Moran’s I = -0.02, SD = 0.01, p = 0.986). Therefore, the results can be treated as not affected by a common evolutionary history of the focal species.

## Discussion

Population trends of birds in the Giant Mts. clearly demonstrate the adverse effect of the climate change on high-altitude species in the past 30 years. It seems that the increasing temperatures, which were observed in the Giant Mts. over the same time period as the bird monitoring was performed, accompanied with the space limitation at high altitudes most likely have detrimental effect on populations of these species. Our results thus provide one of the first robust evidence that the alpine species, which are predicted to undergo serious climatically-induced range contractions in the future due to warming climate [2, 12], already started to move along this trajectory. For example, several studies recognized water pipit, specialist to alpine grasslands, as one of the species being at the highest risk of extinction due to future climatic warming in alpine environments [12, 50], possibly as a consequence of adverse impacts of higher temperatures on species’ physiology, competitive interactions or nest predation rate [50]. Indeed, this species ranks among those with the steepest declines over the study period according to our data providing support for these predictions.

Possible mechanisms of the adverse impacts of warming climate on population of high-altitude species are insufficiently known [51]. Although the habitat composition does not seem to be altered at the highest altitudes in the Giant Mts., warmer climate can cause reduction of food supply for breeding birds, as was observed in the case of red grouse (*Lagopus scoticus*) in the Scottish Highlands [52], or alteration of species interactions, such as increased predation risk on hole-nesting birds due to earlier termination of hibernation in edible dormouse (*Glis glis*) in Moravia [53] or more intensive competition from the side of lower-altitude species [54]. Other factors might include direct detrimental effects of more frequent weather anomalies caused by higher temperatures such as strong storms [55] and physiological stress [56], or complex interactions between climate and local habitat conditions [57]. For example, particularly high temperatures at the start of the spring, which occur since 1990s also in the Giant Mts. [58], can cause rapid melting of snow followed by drought later during the vegetation season, which can constrain food supply in the time of rearing of chicks.

The relationship between migration strategy and bird population trends, when species migrating for longer distances had more negative trends in the Giant Mts. than species migrating for shorter distances, may be also caused by the climate change, although its effect is less clear in this case. Various studies found long-distance migrants to be more sensitive to the climate change impacts due to phenological mismatch [59]. The mismatch occurs when the timing of maximum food supply does not correspond to the time of the highest demands for food during birds’ breeding cycle leading to reduced survival of nestlings and ultimately to population decline [17, 60]. Alternatively, phenological changes in nest cover development can also play a role [61]. However, population consequences of the phenological mismatch need not to be always negative [62]. Moreover, populations of long-distance migrants may also decline due to various other factors such as habitat change at stopover sites and wintering grounds or droughts in Sahel zone [18].

Another possible consequence of climate change impacts on montane species are altitudinal range shifts. By these shifts the species most likely track their climatic optima that moved upward due to the climate change and thus escape the negative population consequences of warming climate [63]. These climatically-induced altitudinal range shifts were observed in montane species throughout the world [5, 6, 8, 64, 65] and birds in the Giant Mts. are among these examples [7]. Therefore, in species breeding at low altitudes, we expected less negative population trends for those showing the longer shifts than for those showing the shorter shifts as a consequence of climatic impacts, and the reverse pattern was our expectation in the case of species breeding at high altitudes. The significant interaction altitudinal range shift × mean altitude of breeding occurrence confirmed these expectations. The mechanism is most likely connected to space limitations near the mountain tops, when the observed shifts are inevitably resulting in range contractions and thus decline in abundance. In contrast, species breeding at lower altitudes have more space to shift upwards. As a consequence, the lower-altitude species with long altitudinal range shifts have less negative trends than long-shifting high-altitude species. They can indeed benefit from milder climate, perhaps due to extended breeding period providing time for more breeding attempts [66], which is hardly possible at high altitudes. These altitudinal differences in the effects of climate change may be accentuated by slightly higher rate of warming at higher altitudes in the Giant Mts., which produces a high pressure on high-altitude birds to shift upwards, but these shifts are not sufficient to track the rapid climate change contributing to population decline [67].

The other traits which were important predictors of interspecific variability in bird population trends in Europe (including the Czech Republic), i.e. life history strategy and European climatic niche [21, 23–25], did not show any significant effects on breeding bird populations in the Giant Mts. We suggest this difference can be explained by specificity of montane conditions differing to some extent from an “average” central European landscape where the data on these traits were collected (see [41] and references therein). As a consequence, the mechanisms which are specific to montane environment probably override the influence of the other drivers which are generally connected with the climate change.

We have to note that the climate change may be not the only driver of the trends in bird populations we detected [68]. Although the land use changes observed in other European mountain ranges such as abandonment of mountain meadows and pastures [69, 70] does not occur in the Giant Mts. [31], we cannot exclude the other factors could limit populations of some species together with climate. Specifically, forest regeneration on sites previously affected by industrial emissions [15] can contribute to the decline of whinchat or tree and meadow pipit [71]. However, land-cover data collected by Corine Land Cover database [72, 73] do not indicate that the proportions of main habitats at the large-scale changed in that direction (expansion of pastures by 14% and reduction of forest cover by 2% between 1990 and 2006). Therefore, we argue that the climate change is the most principal driving force responsible for the patterns in bird populations observed in the Giant Mts. Finally, the climate change *per se* can produce patterns in bird counts due to altered detectability of particular species [74]. Specifically, advancement of species’ arrival due to milder spring temperatures could result in higher counts earlier in the breeding season resulting in overestimates of bird abundance [75]. Although, we cannot exclude that such improved detectability may have contributed to the observed increases in lower-altitude species, this effect acts contra the pattern of declines in high-altitude species over the course of our study.

## Conclusions and Conservation Implications

Our study provides evidence for adverse impacts of current climate change on populations of high-altitude species, here exemplified by birds in the Giant Mts., a central European mountain range. Since recent modelling work predicts for the future more intensive warming on mountains with potentially detrimental impacts on species adapted to the life at mountain tops [2, 12], their negative population trends already found over the last decades are alarming. The fact that the altitudinal range shift further magnifies the differences in trends between the species breeding at lower-altitudes from the trends of the species breeding at high-altitudes, calls for a need of more detailed further studies focusing on causal mechanisms of the impacts of warming climate on particular species. Although knowledge of such mechanisms is crucial for formulating the future conservation strategies, we suggest that it is possible to perform some conservation actions right now focusing on other possible human-induced threats to populations of high-altitude species such as building development, massive tourism and expansion of ski pistes and lifts [76–79]. These threats might contribute to the declines of high-altitude species together with (most likely dominant) climatic changes, but they are easier to mitigate by enforcement of local conservation legislation, especially in protected areas such as the Giant Mts. Along with these options for local actions, we suggest that global scale measures targeted to slow down or even reverse recent climate warming trends are necessary to guarantee the future for the montane species living at the highest altitudes.

## Supporting Information

S1 Table Mean temperatures in the Giant Mountains. The temperatures were measured at three meteorological stations (Labska bouda: 1315 m a.s.l., Pec pod Snezkou: 816 m a.s.l., Janske Lazne: 650 m a.s.l.) from 1980 to 2009.
(DOCX)

S2 Table Population trends and traits of 50 bird species used in the present study. *Trend*–logarithm of mean annual population growth rate with standard error (SE) in the Giant Mountains between 1984 and 2011 computed using log-linear models in TRIM software. *Altitude*–mean altitude of breeding occurrence in the Giant Mts. in 1986–1988 calculated by Reif & Flousek (2012). *Altitudinal range shift*–mean annual shift of breeding occurrence in the Giant Mts. between 1984 and 2011 estimated as a slope of the regression line fitted through mean altitudes of occurrence of a given species in particular years. *Migration strategy*–classification of species according to their migratory behaviour as residents (1), short-distance migrants (2) and long-distance migrants (3) taken from Koleček & Reif (2011). *Life history strategy*–positions of species along a fast-slow life history gradient from “r-selected” to “K-selected” species calculated by Koleček & Reif (2011). *European climatic niche*–mean temperature within species’ European breeding ranges taken from Reif et al. (2013).
(DOCX)

S3 Table Relationships among predictor variables used for the analysis. The relationships were expressed using Pearson correlation coefficient.
(DOCX)
