# Supplementary material for: Population Trends of Central European Montane Birds Provide Evidence for Adverse Impacts of Climate Change on High-Altitude Species
Source: PLoS One. 2015 Oct 1;10(10):e0139465. doi: 10.1371/journal.pone.0139465 (PMC4591356; doi:10.1371/journal.pone.0139465)
Supplement: S1 Table — The temperatures were measured at three meteorological stations (Labska bouda: 1315 m a.s.l., Pec pod Snezkou: 816 m a.s.l., Janske Lazne: 650 m a.s.l.) from 1980 to 2009. (DOCX) [file pone.0139465.s001.docx]

**S1 Table. Mean temperatures in the Giant Mountains.** The temperatures were measured at three meteorological stations (Labska bouda: 1315 m a.s.l., Pec pod Snezkou: 816 m a.s.l., Janske Lazne: 650 m a.s.l.) from 1980 to 2009.

| Year |  | Temperature [°C] |  |
| --- | --- | --- | --- |
|  | Labska bouda | Pec pod Snezkou | Janske Lazne |
| 1980 | 6.1 | 9.8 | 10.9 |
| 1981 | 8.8 | 12.2 | 13.3 |
| 1982 | 8.9 | 12.5 | 13.8 |
| 1983 | 10.1 | 13.5 | 14.8 |
| 1984 | 6.5 | 10.4 | 11.6 |
| 1985 | 8.0 | 11.5 | 12.9 |
| 1986 | 8.9 | 12.6 | 13.9 |
| 1987 | 7.4 | 11.0 | 12.3 |
| 1988 | 9.1 | 12.0 | 13.6 |
| 1989 | 8.5 | 12.0 | 13.2 |
| 1990 | 8.5 | 12.1 | 13.2 |
| 1991 | 7.1 | 10.6 | 11.8 |
| 1992 | 10.0 | 13.2 | 14.3 |
| 1993 | 9.1 | 11.8 | 12.9 |
| 1994 | 10.0 | 12.7 | 14.0 |
| 1995 | 9.4 | 12.4 | 13.3 |
| 1996 | 8.4 | 11.5 | 12.2 |
| 1997 | 9.0 | 12.1 | 13.0 |
| 1998 | 9.3 | 12.4 | 13.3 |
| 1999 | 9.6 | 12.4 | 13.5 |
| 2000 | 10.0 | 12.3 | 13.5 |
| 2001 | 9.6 | 12.1 | 13.1 |
| 2002 | 11.1 | 13.6 | 14.9 |
| 2003 | 11.4 | 14.2 | 14.9 |
| 2004 | 8.1 | 11.4 | 12.1 |
| 2005 | 9.3 | 12.5 | 13.1 |
| 2006 | 10.6 | 14.1 | 14.7 |
| 2007 | 10.2 | 13.1 | 14.3 |
| 2008 | 10.7 | 12.9 | 14.3 |
| 2009 | 8.8 | 11.9 | 13.3 |
