# Supplementary material for: Population Trends of Central European Montane Birds Provide Evidence for Adverse Impacts of Climate Change on High-Altitude Species
Source: PLoS One. 2015 Oct 1;10(10):e0139465. doi: 10.1371/journal.pone.0139465 (PMC4591356; doi:10.1371/journal.pone.0139465)
Supplement: S2 Table — Trend–logarithm of mean annual population growth rate with standard error (SE) in the Giant Mountains between 1984 and 2011 computed using log-linear models in TRIM software. Altitude–mean altitude of breeding occurrence in the Giant Mts. in 1986–1988 calculated by Reif & Flousek (2012). Altitudinal range shift–mean annual shift of breeding occurrence in the Giant Mts. between 1984 and 2011 estimated as a slope of the regression line fitted through mean altitudes of occurrence of a given species in particular years. Migration strategy–classification of species according to their migratory behaviour as residents (1), short-distance migrants (2) and long-distance migrants (3) taken from Koleček & Reif (2011). Life history strategy–positions of species along a fast-slow life history gradient from “r-selected” to “K-selected” species calculated by Koleček & Reif (2011). European climatic niche–mean temperature within species’ European breeding ranges taken from Reif et al. (2013). (DOCX) [file pone.0139465.s002.docx]

**S2 Table. Population trends and traits of 50 bird species used in the present study.**

*Trend* – logarithm of mean annual population growth rate with standard error (SE) in the Giant Mountains between 1984 and 2011 computed using log-linear models in TRIM software. *Altitude* – mean altitude of breeding occurrence in the Giant Mts. in 1986-1988 calculated by Reif & Flousek (2012). *Altitudinal range shift* – mean annual shift of breeding occurrence in the Giant Mts. between 1984 and 2011 estimated as a slope of the regression line fitted through mean altitudes of occurrence of a given species in particular years. *Migration strategy* – classification of species according to their migratory behaviour as residents (1), short-distance migrants (2) and long-distance migrants (3) taken from Koleček & Reif (2011). *Life history strategy* – positions of species along a fast-slow life history gradient from “r-selected” to “K-selected” species calculated by Koleček & Reif (2011). *European climatic niche* – mean temperature within species’ European breeding ranges taken from Reif et al. (2013).

| Species | Trend | SE | Altitude [m] | Altitudinal range shift [m] | Migration strategy | Life history strategy | European climatic niche [°C] |
| --- | --- | --- | --- | --- | --- | --- | --- |
| *Alauda arvensis* | -0.0580 | 0.0099 | 1300 | 4.6 | 2 | -0.93 | 11.7 |
| *Anthus pratensis* | -0.0044 | 0.0071 | 1240 | 3.8 | 2 | -1.08 | 9.9 |
| *Anthus spinoletta* | -0.0327 | 0.0108 | 1324 | -0.1 | 2 | -0.90 | 14.9 |
| *Anthus trivialis* | -0.0353 | 0.0033 | 1049 | 1.4 | 3 | -1.02 | 14.5 |
| *C. coccothraustes* | -0.0052 | 0.6262 | 1085 | -11.5 | 2 | -0.70 | 12.6 |
| *Carduelis cannabina* | 0.0283 | 0.0376 | 1213 | 7.9 | 2 | -1.27 | 12.4 |
| *Carduelis flammea* | -0.0065 | 0.0052 | 1250 | 0.7 | 1 | -1.39 | 12.0 |
| *Carduelis spinus* | 0.0176 | 0.0055 | 975 | 2.6 | 2 | -1.24 | 13.6 |
| *Carpodacus erythrinus* | 0.1163 | 0.2326 | 1159 | 4.4 | 3 | -0.99 | 15.1 |
| *Certhia familiaris* | 0.0201 | 0.0055 | 904 | 0.6 | 1 | -1.31 | 10.6 |
| *Columba palumbus* | 0.0698 | 0.0098 | 875 | 2.5 | 2 | 0.36 | 15.3 |
| *Cuculus canorus* | -0.0037 | 0.0073 | 1030 | 3.5 | 3 | -0.82 | 15.3 |
| *Cyanistes caeruleus* | 0.0568 | 0.0161 | 627 | -0.4 | 1 | -1.42 | 12.1 |
| *Dendrocopos major* | 0.0277 | 0.0095 | 790 | -1.4 | 1 | -0.68 | 15.1 |
| *Dryocopus martius* | -0.0024 | 0.0179 | 942 | 5.9 | 1 | -0.09 | 10.9 |
| *Emberiza citrinella* | -0.0288 | 0.0129 | 842 | 0.6 | 1 | -0.93 | 11.2 |
| *Erithacus rubecula* | 0.0004 | 0.0026 | 974 | -0.5 | 2 | -1.11 | 11.6 |
| *Ficedula hypoleuca* | 0.0225 | 0.0150 | 892 | 1.9 | 3 | -1.07 | 13.8 |
| *Fringilla coelebs* | -0.0092 | 0.0013 | 1014 | -0.5 | 2 | -1.06 | 11.8 |
| *Garrulus glandarius* | 0.0349 | 0.0082 | 902 | 2.3 | 1 | -0.22 | 11.8 |
| *Lanius collurio* | 0.1240 | 0.3442 | 893 | 3.6 | 3 | -0.70 | 15.5 |
| *Lophophanes cristatus* | 0.0182 | 0.0120 | 942 | -1.3 | 1 | -1.15 | 11.4 |
| *Loxia curvirostra* | 0.0098 | 0.0069 | 1008 | 0.2 | 2 | -0.80 | 1.4 |
| *Luscinia svecica* | -0.0147 | 0.0095 | 1376 | 0.8 | 2 | -1.02 | 10.0 |
| *Motacilla alba* | -0.0096 | 0.0165 | 1093 | 10.0 | 2 | -1.11 | 11.6 |
| *Motacilla cinerea* | 0.0026 | 0.0039 | 1041 | 0.1 | 2 | -1.22 | 12.4 |
| *Nucifraga caryocatactes* | 0.0392 | 0.1427 | 998 | 1.8 | 1 | -0.03 | 6.0 |
| *Parus major* | 0.0072 | 0.0089 | 780 | -0.4 | 1 | -1.31 | 11.8 |
| *Periparus ater* | 0.0278 | 0.0036 | 961 | -1.0 | 1 | -1.37 | 11.6 |
| *Phoenicurus ochruros* | -0.0709 | 0.0101 | 1185 | 5.6 | 2 | -1.02 | 12.7 |
| *Phoenicurus phoenicurus* | -0.0047 | 0.0085 | 1041 | 0.4 | 3 | -1.15 | 14.7 |
| *Phylloscopus collybita* | 0.0232 | 0.0033 | 994 | 2.0 | 2 | -1.37 | 11.5 |
| *Phylloscopus sibilatrix* | -0.0174 | 0.0076 | 857 | -0.6 | 3 | -1.25 | 14.6 |
| *Phylloscopus trochilus* | 0.0380 | 0.0064 | 1057 | 2.5 | 3 | -1.28 | 10.2 |
| *Prunella modularis* | 0.0220 | 0.0027 | 1034 | 1.1 | 2 | -1.13 | 10.8 |
| *Pyrrhula pyrrhula* | 0.0325 | 0.0057 | 944 | -0.8 | 1 | -0.89 | 10.7 |
| *Regulus ignicapilla* | 0.0380 | 0.0043 | 883 | 1.6 | 2 | -1.55 | 15.9 |
| *Regulus regulus* | 0.0110 | 0.0020 | 960 | -0.4 | 1 | -1.57 | 10.7 |
| *Saxicola rubetra* | -0.0138 | 0.0179 | 1227 | 8.1 | 3 | -1.04 | 14.4 |
| *Sitta europaea* | 0.0462 | 0.0129 | 826 | -1.8 | 1 | -0.90 | 12.4 |
| *Streptopelia turtur* | 0.0193 | 0.0280 | 780 | 6.1 | 3 | 0.00 | 16.5 |
| *Sylvia atricapilla* | 0.0567 | 0.0041 | 932 | 5.2 | 2 | -1.11 | 15.7 |
| *Sylvia borin* | -0.0466 | 0.0148 | 839 | 0.3 | 3 | -1.09 | 14.5 |
| *Sylvia communis* | 0.0483 | 0.0119 | 1006 | 4.4 | 3 | -1.12 | 15.6 |
| *Sylvia curruca* | -0.0077 | 0.0168 | 946 | 7.6 | 3 | -1.26 | 14.8 |
| *Troglodytes troglodytes* | 0.0342 | 0.0033 | 950 | 0.6 | 1 | -1.21 | 15.4 |
| *Turdus merula* | 0.0343 | 0.0057 | 868 | 3.3 | 1 | -0.50 | 12.0 |
| *Turdus philomelos* | -0.0079 | 0.0032 | 940 | 0.4 | 2 | -0.70 | 11.1 |
| *Turdus torquatus* | 0.0410 | 0.0119 | 1212 | 0.0 | 2 | -0.34 | 13.0 |
| *Turdus viscivorus* | 0.0111 | 0.0047 | 962 | -1.0 | 2 | -0.34 | 7.3 |
