# Supplementary material for: Population Trends of Central European Montane Birds Provide Evidence for Adverse Impacts of Climate Change on High-Altitude Species
Source: PLoS One. 2015 Oct 1;10(10):e0139465. doi: 10.1371/journal.pone.0139465 (PMC4591356; doi:10.1371/journal.pone.0139465)
Supplement: S3 Table — The relationships were expressed using Pearson correlation coefficient. (DOCX) [file pone.0139465.s003.docx]

**S3 Table. Relationships among predictor variables used for the analysis.** The relationships were expressed using Pearson correlation coefficient.

|  | Altitude | Altitudinal range shift | Migration strategy | Life history strategy |
| --- | --- | --- | --- | --- |
| Altitudinal range shift | 0.20 |  |  |  |
| Migration strategy | 0.22 | 0.32 |  |  |
| Life history strategy | -0.10 | 0.05 | -0.04 |  |
| European climatic niche | -0.12 | 0.20 | 0.40 | -0.09 |
